# ELLI-1, a novel germline protein, modulates RNAi activity and P-granule accumulation in *Caenorhabditis elegans*

**DOI:** 10.1371/journal.pgen.1006611

**Published:** 2017-02-09

**Authors:** Karolina M. Andralojc, Anne C. Campbell, Ashley L. Kelly, Markus Terrey, Paige C. Tanner, Ian M. Gans, Michael J. Senter-Zapata, Eraj S. Khokhar, Dustin L. Updike

**Affiliations:** The Mount Desert Island Biological Laboratory, Bar Harbor, Maine, United States of America; The University of North Carolina at Chapel Hill, UNITED STATES

## Abstract

Germ cells contain non-membrane bound cytoplasmic organelles that help maintain germline integrity. In *C*. *elegans* they are called P granules; without them, the germline undergoes partial masculinization and aberrant differentiation. One key P-granule component is the Argonaute CSR-1, a small-RNA binding protein that antagonizes accumulation of sperm-specific transcripts in developing oocytes and fine-tunes expression of proteins critical to early embryogenesis. Loss of CSR-1 complex components results in a very specific, enlarged P-granule phenotype. In a forward screen to identify mutants with abnormal P granules, ten alleles were recovered with a *csr-1* P-granule phenotype, eight of which contain mutations in known components of the CSR-1 complex (*csr-1*, *ego-1*, *ekl-1*, and *drh-3)*. The remaining two alleles are in a novel gene now called *elli-1* (enlarged germline granules). ELLI-1 is first expressed in primordial germ cells during mid-embryogenesis, and continues to be expressed in the adult germline. While ELLI-1 forms cytoplasmic aggregates, they occasionally dock, but do not co-localize with P granules. Instead, the majority of ELLI-1 aggregates accumulate in the shared germline cytoplasm. In *elli-1* mutants, several genes that promote RNAi and P-granule accumulation are upregulated, and embryonic lethality, sterility, and RNAi resistance in a hypomorphic *drh-3* allele is enhanced, suggesting that ELLI-1 functions with CSR-1 to modulate RNAi activity, P-granule accumulation, and post-transcriptional expression in the germline.

## Author summary

Germ cells are unique because of their immortal potential, giving rise to gametes that fertilize and become the next generation. This immortal potential is regulated in both the nucleus and cytoplasm. Within the cytoplasm are special ‘germ granules’, consisting of a heterogeneous mix of proteins and RNA, that provide a microenvironment for germline specific post-transcriptional processes. Germ granules are readily observed in living worms, making *C*. *elegans* a useful model to study germ-granule function. Recently, the Argonaute protein CSR-1 was found to be central to germ-granule function; loss of known components of the CSR-1 complex cause a distinctive enlarged germ-granule phenotype. Here we describe an unbiased approach using this phenotype to identify additional mutations in known CSR-1 complex components, along with mutations in a previously undescribed gene we’ve called *elli-1*. Germline defects associated with compromising the CSR-1 complex are enhanced by *elli-1*, suggesting that ELLI-1 cooperates with this complex and a provides a key to deciphering how CSR-1 functions in germ granules to promote germ cell integrity.

## Introduction

P granules are required in the *C*. *elegans* adult germline to maintain fertility and protect germ cell fate [1]. Recent studies confirm the overlap between P-granule function and siRNA regulation. PGL-1, which nucleates P-granule formation, is RNAi defective (Rde) [2]; while VASA/GLH-1, another constitutive P-granule component, interacts directly with DCR-1 to regulate P-granule structure [3]. The Argonaute CSR-1 is also recruited to P granules through the Dicer-Related Helicase DRH-3, the RNA-Dependent RNA Polymerase (RDRP) EGO-1, and a Tudor-Domain protein called EKL-1 [4]. DRH-3, EGO-1, EKL-1, and CSR-1 constitute the CSR-1 22G RNA complex and each are essential for fertility and efficient RNAi [5–10], though it is unknown whether this RNAi defect is direct or an indirect consequence of having enlarged P granules and compromised PGL-1 [4,11,12]. Determining the cause and the consequence of the Rde phenotype in P-granule assembly and CSR-1 pathway mutants is a challenge because both types of mutations affect P-granule structure and morphology.

Recently, mRNA-seq of dissected P-granule and *csr-1* depleted germlines revealed a strong correlation in regulated transcripts, with the majority of upregulated genes in both P-granule and *csr-1* depleted germlines found in sperm-enriched datasets [13]. This is accompanied by the distal expansion of sperm transcripts into what become intersexual germ cells that rarely fertilize. This partial masculinization of developing oocytes after depletion of *csr-1* is likely explained by a 9.5-fold overexpression of *fog-3*, the effector of the germline sex determination pathway [13]. A separate more recent report profiled gene expression changes in whole worms where CSR-1’s enzymatic RNA slicing activity was inactivated, which also found significant enrichment in sperm transcripts and a 13.9-fold increase in *fog-3* expression; however, worms with inactive CSR-1 slicer activity have somewhat higher broods than *csr-1* deletions and no reported P-granule phenotype [14]. These observations may suggest that CSR-1’s slicing activities repress masculinization of developing oocytes while CSR-1’s slicer independent functions maintain P-granule integrity. These slicer-independent functions could involve translational regulation, which cannot be directly assessed in genome-wide RNA expression studies [15]. In addition to antagonizing sperm-specific transcripts in developing oocytes [5,13], P-granule assembly [4,11,12], and small RNA biogenesis [4,16–18], CSR-1 and its cofactors have also been implicated in mitosis and meiosis [7–9,19,20], transcription [21], chromatin condensation and kinetochore assembly [4,22–24], H3K9me2 distribution [22,25], histone and histone mRNA maturation [26], alternative splicing [27], and the epigenetic licensing of germline transcripts for expression [28–34]. While some of these roles will likely be more direct than others, the importance of CSR-1 in the *C*. *elegans* germline cannot be understated, and finding additional factors that interact with the CSR-1 complex is imperative to parsing out its functions.

Here we report results of a forward mutagenesis for regulators of P-granule assembly. Using this unbiased approach, we identified a class of enlarged P-granule mutants harboring loss-of-function alleles of all four known CSR-1 complex components, in addition to loss-of-function mutations in a novel gene we’ve named *elli-1* (enlarged germline granules). We also describe ELLI-1 expression in the germline, differential gene expression in *elli-1* mutants, and *elli-1*’s genetic interaction with the CSR-1 pathway.

## Results

### Forward genetic screen identifies mutants with enlarged P granules

**Fig 1:**
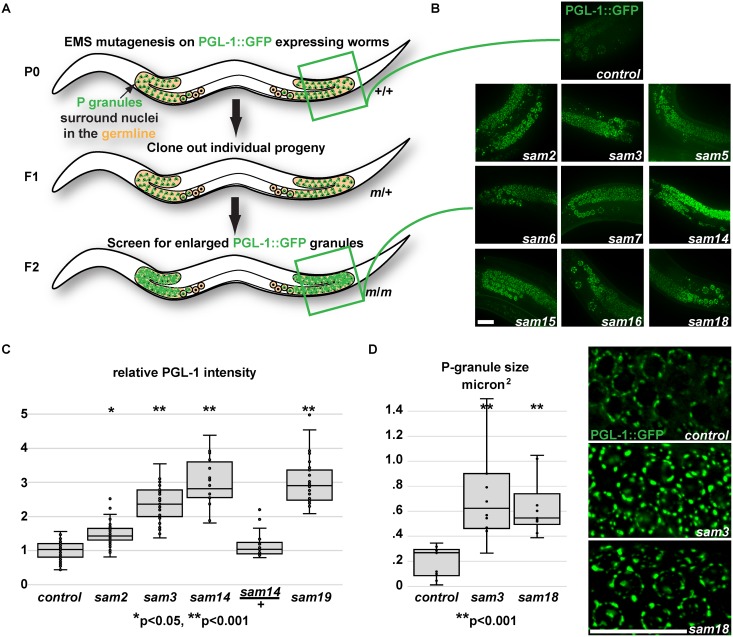
Screen for regulators of P-granule accumulation. A) Screening strategy to isolate mutants with enlarged PGL-1::GFP granules. PGL-1::GFP worms were mutagenized, 2000 F1 progeny were cloned to individual plates, and F2 progeny were screened for homozygous (m/m) mutants with enlarged P granules B) PGL-1::GFP expression in the gonad arm of parental (P0) and mutant strains. C) Relative PGL-1 intensity in mutant germlines normalized to the parental control. D) Increased PGL-1::GFP size in mutants correlates with increased PGL-1 intensity.

To discover regulators of P-granule accumulation, EMS mutagenesis was performed on a *C*. *elegans* strain with an integrated transgene expressing the constitutive P-granule component, PGL-1, tagged with GFP (Fig 1A) [35]. The F2 generation was then screened for accumulation of large and more intense PGL-1::GFP granules (Fig 1B), similar to those previously observed with *csr-1* RNAi [12]. Ten independent, fully-penetrant alleles were isolated, and single nucleotide polymorphisms mapped six alleles to three complementation groups on chromosome I, and four alleles to two complementation groups on chromosome IV (Table 1). PGL-1 intensity was imaged under fixed exposure conditions and quantified, with *sam2* exhibiting the smallest increase in PGL-1 intensity to *sam14* and *sam19* exhibiting the highest intensity (Fig 1C). A closer comparison of P-granule size between *wild-type*, *sam3*, and *sam18* shows that P granules are two to three times larger in *sam3* and *sam18* mutants, suggesting that the observed increase in intensity reflects both larger P granules and more abundant PGL-1::GFP (Fig 1D).

**Table 1:** Enlarged P-granule alleles from screen.

Allele	Chromosome	Fails to Complement	Gene	Sterile
*sam2*	I	*sam5*	*drh-3*	temp sensitive sterile
*sam3*	IV	*sam6*	*elli-1*	no
*sam5*	I	*sam2*	*drh-3*	homozygous sterile
*sam6*	IV	*sam3*	*elli-1*	no, but linked to Emb
*sam7*	I	*sam14*, *sam16*	*ego-1*	homozygous sterile
*sam14*	I	*sam7*, *sam16*	*ego-1*	homozygous sterile
*sam15*	IV	*sam18*, *csr-1(fj54)*	*csr-1*	homozygous sterile
*sam16*	I	*sam7*, *sam14*	*ego-1*	homozygous sterile
*sam18*	IV	*sam15*, *csr-1(fj54)*	*csr-1*	homozygous sterile
*sam19*	I		*ekl-1*	homozygous sterile

**Fig 2:**
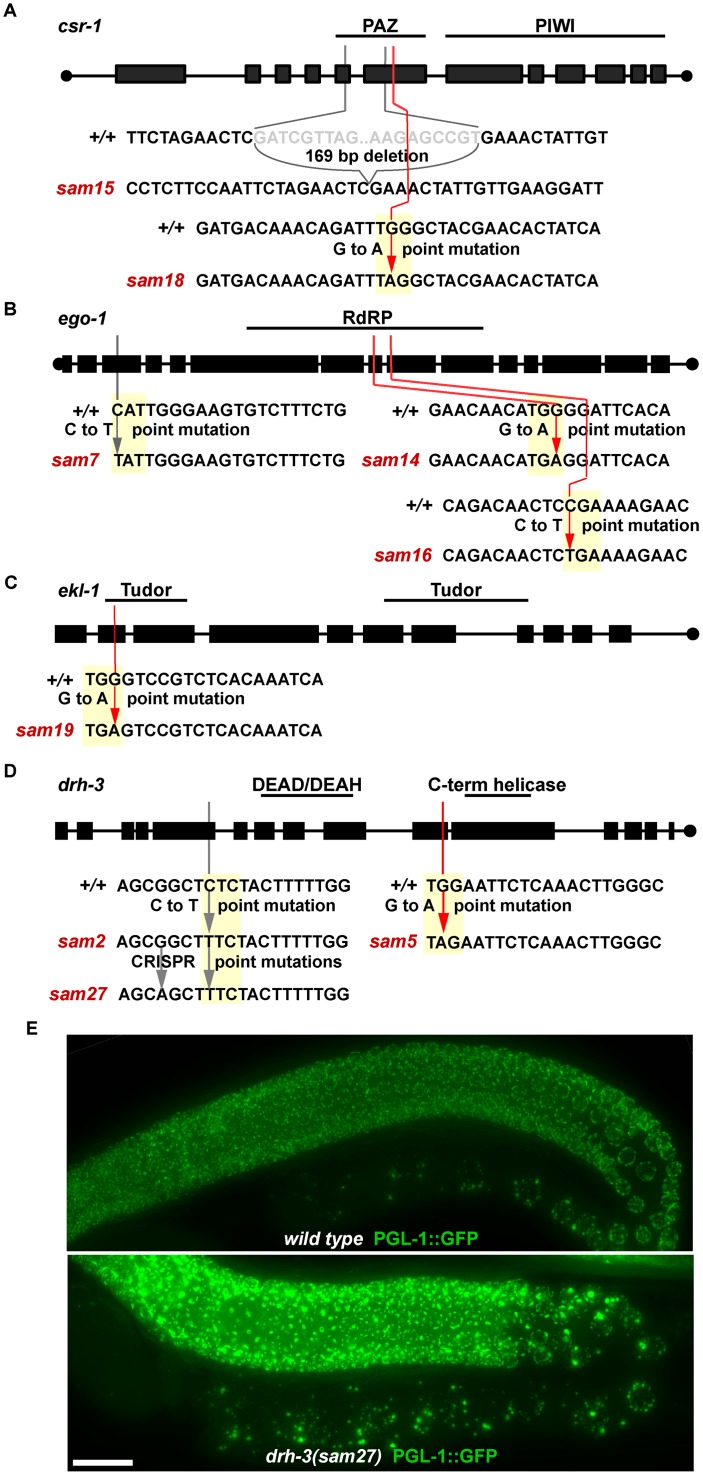
Isolation of new CSR-1 complex alleles. A-D) Location and flanking sequences for new alleles of CSR-1 complex components. Red lines show early stop codons, grey lines show base pair substitutions. Yellow boxes indicate a codon in the reading frame. E) Endogenous PGL-1::GFP expression in *wild-type* and *drh-3(sam27)* germlines.

To determine if alleles mapping to chromosome IV contain mutations in *csr-1/F20D12*.*1*, complementation tests were performed with *csr-1(fj54)*. Large and bright P granules were observed in both *csr-1(fj54)/sam15* and *csr-1(fj54)/sam18* cross progeny, showing that these alleles failed to complement, while P granules in *csr-1(fj54)/sam3* and *csr-1(fj54)/sam6* cross progeny were normal (n>15 progeny examined from each cross). Sequencing *sam15* revealed a 169 base pair deletion in the PAZ domain of *csr-1* that causes a frameshift starting at amino acid 384 of *F20D12*.*1a*, and an early stop codon 14 amino acids later (Fig 2A). Sequencing *sam18* revealed a G to A point mutation in *csr-1*’s PAZ domain that causes an early stop codon at amino acid 443 of *F20D12*.*1a*. Like other *csr-1* alleles, *sam15* and *sam18* have very small broods of early arrested embryos and must be maintained over a balancer. The isolation of two *csr-1* alleles from this screen demonstrates the specificity of the large P-granule accumulation phenotype, making it likely that this screen isolated alleles of known CSR-1 co-factors.

Hawaiian Variant Mapping was used in combination with genome-wide sequencing and the Cloudmap pipeline to identify the remaining alleles on chromosomes I and IV [35,36]. On chromosome I, mutations were identified in three known *csr-1* cofactors. Both *sam14* and *sam16* contained point mutations that introduced stop codons in the RNA dependent RNA polymerase (RdRP) domain of *ego-1*, while *sam7* contained an N-terminal missense mutation in the same gene (Fig 2B), and all three alleles are homozygous sterile. *sam19* contains a point mutation that introduces an early stop codon in the first Tudor domain of *ekl-1*, and is also homozygous sterile (Fig 2C). *sam5* contains a G to A point mutation that introduces an early stop codon in exon 10 of *drh-3*, while in *sam2* a C to T missense substitution (Leu307Phe) was discovered in exon 5 of the same gene. This mutation lies directly before the DEAD/DEAH helicase coding domains of *drh-3* and causes a relatively minor change, reflecting the subtler phenotype of *sam2* homozygotes, which are only sterile when raised at a higher (25°C) temperature (Fig 2D). CRISPR was used to evaluate whether this simple substitution of hydrophobic residues was sufficient to cause a P-granule phenotype. A C to T base pair change recapitulating the original mutation plus a silent mutation to prevent Cas9 re-cleavage [*drh-3(sam27)*] was introduced in wild-type worms, which were then crossed into a new CRISPR-derived *pgl-1(sam33[pgl-1*::*gfp*::*3xFLAG])* line. *drh-3(sam27)* reproduced the enlarged PGL-1::GFP expression, confirming that this missense mutation is responsible for the P-granule phenotype of *drh-3(sam2)* (Fig 2E).

**Fig 3:**
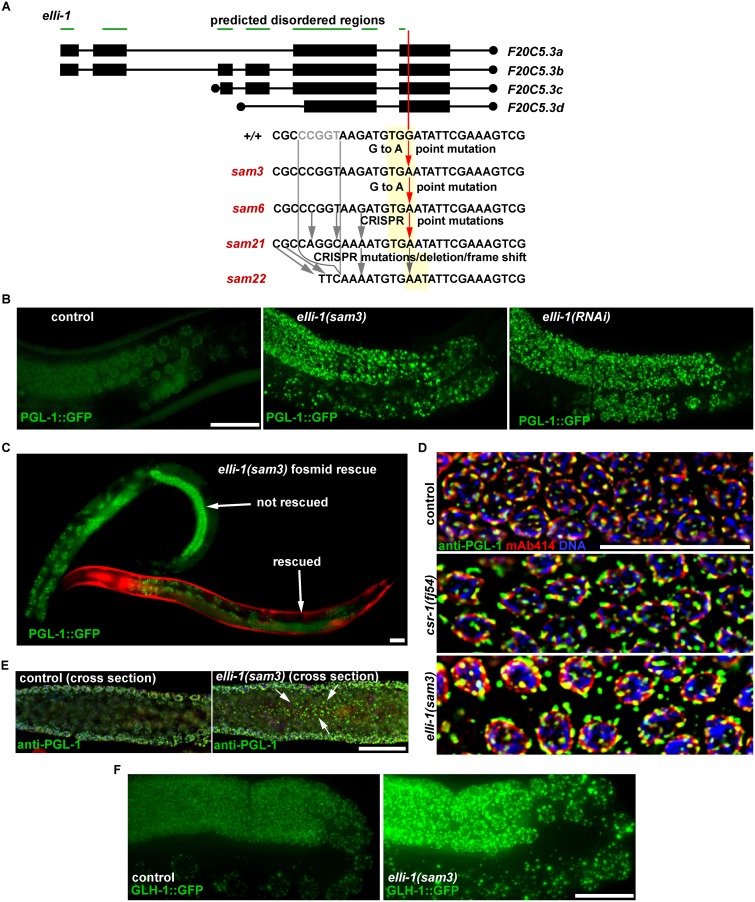
*elli-1* alleles and phenotypes. A) Location and flanking sequences of *elli-1* alleles. Red lines show early stop codons, grey lines show base pair substitutions and deletions. Yellow boxes indicate a codon in the reading frame. ELLI-1’s N-terminal predicted disordered region is shown by the green bars. B) *elli-1(sam3)* and *elli-1(RNAi)* cause bright expression of enlarged PGL-1::GFP granules. C) Fosmid rescue of bright PGL-1::GFP expression in *elli-1(sam3)* animals. Red body-wall muscles mark animals with the rescuing fosmid. D) Cross-section through pachytene germ cells on the surface of wild-type and *elli-1(sam3)* germlines stained with anti-PGL-1 (green), anti-Nuclear Pore Complex antibody mAb414 (red), and DAPI/DNA (blue). P granules in *csr-1* and *elli-1* germlines are enlarged and often detach from the nuclear periphery. E) Cross-section through *wild-type* and *elli-1* germlines with the same strains as in D. Arrows show PGL-1 accumulation in the shared cytoplasm. F) GFP tagging endogenous GLH-1 in germlines of *wild-type* and *elli-1(sam3)* worms.

Because this screen identified multiple alleles of *csr-1* and its cofactors *ego-1*, *ekl-1*, and *drh-3*, other mutations with identical P-granule phenotypes likely interact with the same complex. Only one complementation group consisting of *sam3* and *sam6* remained. Following the sequencing strategy above, it was discovered that both alleles contain a G to A point mutation that causes an early stop codon in the last exon of *F20C5*.*3* (Trp→Stop at amino acid 184 of *F20C5*.*3a*, Fig 3A). This mutation is not found in the parental strain or the *csr-1*, *ego-1*, *ekl-1* or *drh-3* mutant strains. Given that *sam3* and *sam6* were isolated from different F1s following mutagenesis, these two alleles may have arisen in pre-meiotic germ cells from the same mutagenized animal. However, because of a closely linked lethal mutation in *sam6*, it cannot be passaged as a homozygous strain like *sam3*. Targeting *F20C5*.*3* with RNAi causes bright, enlarged P granules (in 124/140 RNAi fed worms) (Fig 3B), and injection of a fosmid carrying *F20C5*.*3* rescues the P-granule phenotype in *sam3* mutants (36/38 transgenic animals rescued) (Fig 3C). *F20C5*.*3* has now been renamed *elli-1*, for its enlarged germline granules.

The PGL-1::GFP reporter used in the screen is likely a low copy transgene integrated on chromosome I; however, the actual copy number is unknown. To ensure that the enlarged and bright P-granule phenotype of *elli-1(sam3)* was not simply de-silencing the integrated transgene, PGL-1::GFP was crossed out of *elli-1(sam3)* worms and endogenous PGL-1 was imaged with a PGL-1 antibody (Fig 3D). The size and intensity of PGL-1 granules were higher in *elli-1(sam3)* compared to the control (p = 0.0001) and indistinguishable from those in *csr-1(fj54)* germlines (Fig 3D, representative images of 10 fixed and stained germlines for each condition). In both *elli-1(sam3)* and *csr-1(fj54)* germlines, some PGL-1 granules were observed to detach from the nuclear envelope (mAb414 stain in red), likely contributing to the accumulation of excess PGL-1::GFP and endogenous PGL-1 granules in the rachis of elli-1 mutants (Fig 3E, 8/8 *elli-1* compared to 0/8 N2, p = 0.0002). This detachment may be caused by the spherical shape of larger granules, decreasing the surface in contact with the nuclear periphery. Worms lacking the GFP transgene were then crossed into a new CRISPR-derived *glh-1(sam24[glh-1*::*gfp*::*3xFLAG])* line. The size and intensity of endogenous GLH-1 tagged with GFP also increased in germ cells of *elli-1(sam3)* mutants, showing that the increase in P-granules size is not specific to PGL-1 (Fig 3F). Constructs were generated to re-introduce the *elli-1(sam3)* point mutation into the GFP::PGL-1 transgenic strain using CRISPR. From this experiment, two lines were generated (Fig 3A): one with the desired point mutation plus three silent mutations to prevent Cas9 re-cleavage [*elli-1(sam21)*], and one complex rearrangement near the mutated base [*elli-1(sam22)*]. The complex rearrangement in *elli-1(sam22)* causes a Pro→Leu mutation at 179aa of F20C5.3a, a frameshift at 180aa, and a stop codon 34 aa later. Both *elli-1(sam21)* and *elli-1(sam22)* exhibit a fully-penetrant and identical P-granule phenotype to *elli-1(sam3)* and *elli-1(sam6)*. Taken together, these results demonstrate that *elli-1/F20C5*.*3* loss-of-function causes the accumulation of large P granules in the *C*. *elegans* germline.

### ELLI-1 is a novel germline component that functions in early development

**Fig 4:**
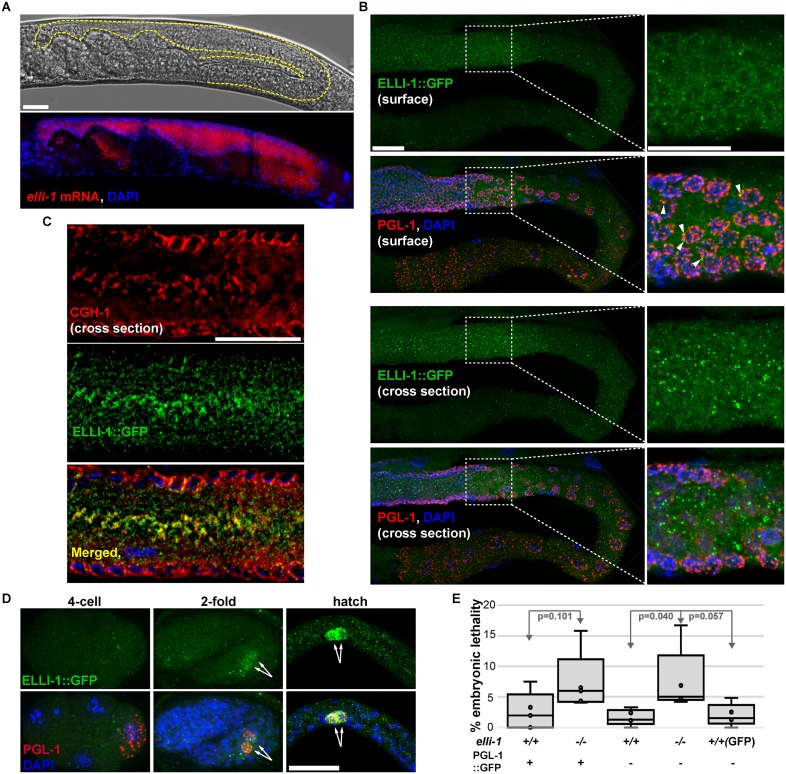
*elli-1* expression. A) Fluorescence In Situ Hybridization (FISH) of *elli-1* mRNA (red) shows germline expression. DAPI/DNA is blue. B) *elli-1*::*GFP*::*3xFLAG* showing diffuse cytoplasmic expression with some ELLI-1 foci in a dissected germline. 4.3% of ELLI-1 foci are docked to P granules (arrowheads), but most of these foci are in the central shared cytoplasm of the rachis. C) ELLI-1::GFP partially overlaps with the expression of the P-body component CGH-1 (red), primarily in the rachis instead of at the nuclear periphery of germ cells. D) *elli-1*::*GFP*::*3xFLAG* expression in primordial germ cells (arrows) during embryogenesis and the first larval stage. E) Embryonic lethality associated in *elli-1* worms.

ELLI-1 is a nematode-specific protein with no discernable domains. MobiDB predicts that most of ELLI-1 contains disordered regions (Fig 3A, green bars) [37,38]. RNA-seq of dissected germlines shows *elli-1* transcripts are expressed more abundantly than 91% of genes [13], and expression profiling in germline-less animals previously showed that *elli-1* is germline enriched [39,40]. To visualize *elli-1* transcripts in whole worms, fluorescent in situ hybridization (FISH) was performed [13], showing that *elli-1* mRNA is indeed enriched in the germline (Fig 4A). To observe the sub-cellular localization of ELLI-1 protein, CRISPR was used to place a C-terminal GFP-3xFLAG tag on endogenous *elli-1* [41]. A diffuse GFP signal can be observed at low levels in the germline of these living worms, and M2 FLAG antibody shows diffuse ELLI-1 expression in germline cytoplasm with some small ELLI-1 foci (Fig 4B). While 4.3% of these foci can be found docked next to P granules (Fig 4B, arrowheads), the majority of ELLI-1 foci accumulate in the rachis (averaged from 10 germlines). Within the rachis, ELLI-1 foci partially overlap with the P-body component CGH-1 (Fig 4C), suggesting these foci are associated with RNA. However, ELLI-1’s RNA-binding affinity has yet to be examined and may be indirect. We sought to determine whether ELLI-1 colocalizes with PATR-1, a P-granule component found only in P bodes but not P granules, but an available PATR-1 antibody did not work in dissected germlines, and a CRISPR-derived PATR-1::GFP strain generated for this study was surprisingly diffuse in the adult germline and did not form P-body-like foci. Zygotic ELLI-1 begins to accumulate in the cytoplasm of primordial germ cells between the comma to 2-fold stage of embryogenesis, but again appears distributed throughout the germ plasm instead of concentrated on P granules (Fig 4D).

In an RNAi screen of oocyte-enriched transcripts, it was previously discovered that depletion of *elli-1* caused partially penetrant embryonic lethality, suggesting a role in early development [42]. An early embryonic arrest (30–100 cell stage) trend was observed in 5–8% of *elli-1* worms, confirming this role (Fig 4E). Embryonic lethality and P-granule phenotypes are not observed above background in the *elli-1*::GFP strain, suggesting that the GFP tag is not compromising ELLI-1 function.

### ELLI-1 modulates transcripts encoding core P-granule and RNAi components

**Fig 5:**
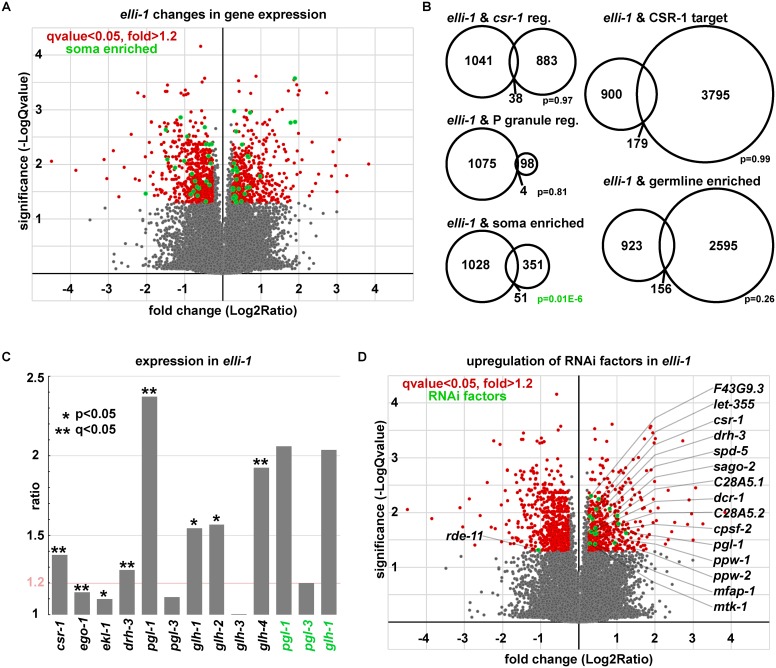
Gene expression analysis of *elli-1*. A) Volcano plot showing the fold change and significance of gene expression in *elli-1*. Significantly regulated genes above or below 1.2-fold are shown in red, with a subset of soma-enriched genes [43] in green (see S1 Table). B) Proportional Venn diagrams comparing overlap between the 1079 *elli-1* regulated genes and previously published *csr-1* and *pgl-1* regulated [13], CSR-1 target [4], and soma and germline enriched [43] datasets. C) Increased average expression of CSR-1 complex and core P-granule components in the *elli-1* expression array (black) and *elli-1* qRTPCR (green). Pink line indicates the arbitrary 1.2-fold increased expression cutoff. Increased *ego-1* and *ekl-1* expression was statistically significant, but under the 1.2-fold cutoff. D) *elli-1* Volcano plot showing increased expression of genes required for RNAi-dependent gene silencing (green).

To gain insight into the function of ELLI-1, tiling arrays were used to compare the whole-worm RNA expression profile of *elli-1(sam3)* to wild-type animals (four biological replicates each). This analysis showed significant changes (over 1.2-fold, q value < 0.05) in expression for 1079 genes, 43% of which are upregulated while 57% are downregulated (Fig 5A red, S1 Table). No significant overlap was observed when comparing these 1079 genes to previously published datasets of *csr-1* regulated genes [13], *pgl-1* regulated genes [13], CSR-1 target genes [4], or germline enriched genes [43] (Fig 5B). There was a statistically significant overlap of genes that are soma-enriched [43], but they were evenly distributed between genes that were up and downregulated in the *elli-1* mutant (Fig 5A green, Fig 5B). Expression of transcripts encoding CSR-1 and its cofactors EGO-1, EKL-1, and DRH-3 showed modest but significant increases in *elli-1* (Fig 5C). Consistent with the enlarged P-granule phenotype of *elli-1*, transcripts encoding the constitutive P-granule components PGL-1, GLH-1, GLH-2, and GLH-4 increased in the mutant (2.37, 1.54, 1.57, and 1.92 fold respectively); although, this increase was not seen with all P-granule transcripts as those encoding PGL-3 and GLH-3 changed very little (Fig 5C). Quantitative RT-PCR on *pgl-1*, *pgl-3*, and *glh-1* in wild-type and an outcrossed *elli-1(sam3)* line validated the microarray results (Fig 5C, green). As P granules regulate translational silencing in the germline, increased expression of P-granule components suggest that one function of ELLI-1 may be to keep P-granule accumulation and translational silencing in check; however, an alternative interpretation is also discussed below.

**Fig 6:**
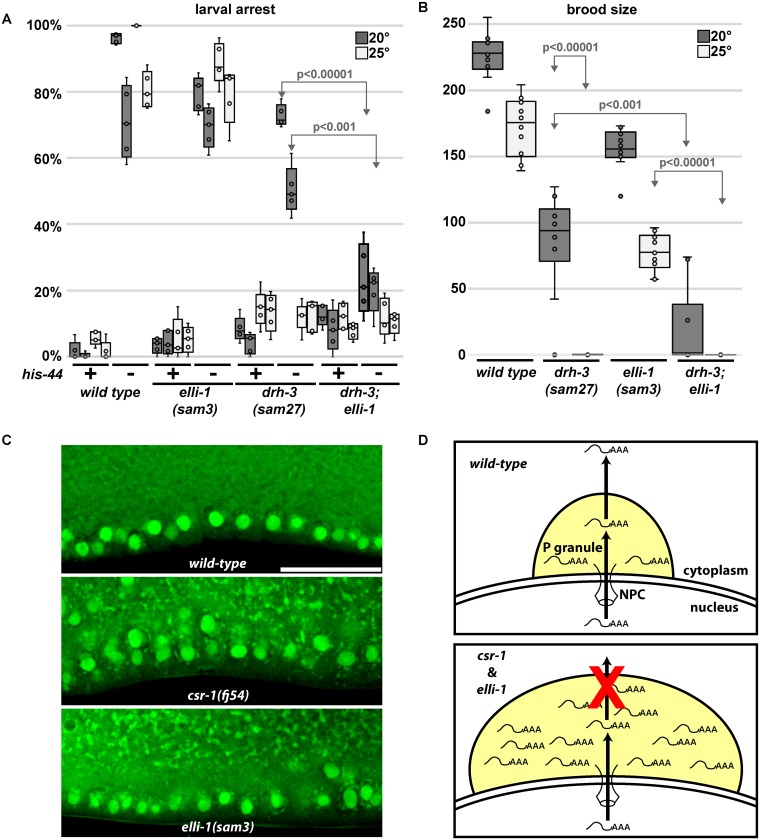
Synthetic sterility and RNAi deficiency in *elli-1*. A) Larval arrest on control (+) and *his-44* (-) RNAi showing *elli-1(sam3)* enhances the RNAi resistant phenotype of *drh-3(sam27)* at permissive temperature (20°C). Same colored box plots represent duplicate experiments performed on different weeks. B) Brood sizes show that *elli-1(sam3)* enhances sterility (no brood) of *drh-3(sam27)* at permissive temperature. C) STYO14 staining of dissected germlines shows RNA pooling around the nuclear periphery and in the rachis of both *csr-1* and *elli-1* mutants. D) Model for CSR-1 and ELLI-1 function. P granules (yellow) reside on the nuclear periphery where they receive transcripts coming through the nuclear pore complex. The CSR-1 complex functions in P granules to recognize germline abundant or licensed transcripts. Our model is that CSR-1 and ELLI-1 function to move germline abundant or licensed transcripts through and away from P granules and into the cytoplasm. In mutants this dispersal of RNA is blocked, RNA and P granules accumulate, and fertility and RNAi efficacy is decreased. It remains unclear whether ELLI-1 directly interacts with RNA.

Gene ontology analysis of the 1079 transcripts with changed expression in *elli-1(sam3)* mutants revealed an enrichment in genes required for RNAi-dependent gene silencing [2,6–9,44–46] (See S1 Table). All but one (*rde-11*) of 16 significantly regulated RNAi genes are overexpressed in *elli-1* animals (Fig 5D, green). These genes encode the core RNAi factor DCR-1, the DEAD/DEAH helicase DRH-3, four RNAi-dependent Argonautes (CSR-1, PPW-1, PPW-2, and SAGO-2), and ten other genes with endogenous and exogenous RNAi defects [6–9,44,45,47,48]. Because of the abundant overexpression of RNAi factors in the *elli-1* mutant, *his-44* RNAi feeding was used to examine enhanced or suppressed larval arrest phenotypes in the *elli-1* background. *his-44* RNAi causes a larval arrest phenotype and can be used to determine RNAi efficiency [49]. In the *elli-1(sam3)* background alone there was no significant difference in the *his-44* RNAi response when compared to wild-type worms (Fig 6A). Therefore, increased expression of RNAi components in *elli-1* worms is a likely reflection of enlarged P granules, where several RNAi components reside, but does not enhance or suppress RNAi sensitivity by itself.

### ELLI-1 enhances sterility and RNAi-defective phenotypes when the CSR-1 complex is partially compromised

Similarities between the P-granule phenotypes of *csr-1*, *ego-1*, *ekl-1*, *drh-3* and *elli-1* suggests ELLI-1 functions with or in parallel to CSR-1; however, unlike *elli-1*, null mutations in CSR-1 complex components are RNAi defective and have no viable progeny [4]. This difference, and the lack of correlation between differentially expressed genes in *elli-1* and *csr-1* worms, may suggest the P-granule phenotypes are coincidental; however, given the role of P granules in translational regulation, mutations in CSR-1 complex components may still interact *genetically* with *elli-1*. To see if there is a genetic interaction, the homozygous viable *drh-3(sam27)* allele was utilized. Hypomorphic alleles of *drh-3* previously isolated from an RNAi-deficiency screen have viable progeny at permissive temperature, but are sterile at elevated temperatures [10]. First, broods were counted from individual *drh-3(sam27)* animals grown at 20°C and 25°C, showing this new allele is also temperature sensitive sterile (Fig 6B). To see if *elli-1(sam3)* enhanced sterility of *drh-3(sam27)*, brood sizes from individual worms were then counted in the *drh-3(sam27); elli-1(sam3)* double mutant. At the permissive temperature of 20°C, *drh-3(sam27)* and *elli-1(sam3)* single mutants are fertile, but the majority of double mutants are not. This double mutant line must be maintained at 15°C, as the small percentage of fertile animals could not be passaged beyond three generations at 20°C. RNAi sensitivity was also tested in the double mutant (Fig 6A). *drh-3(sam27)* animals suppress larval arrest when fed *his-44* RNAi at 25°C, but only partially suppress larval arrest at 20°C. In contrast, the *drh-3(sam27); elli-1(sam3)* double mutant suppresses *his-44* RNAi larval arrest at both permissive and restrictive temperatures. Thus, *elli-1(sam3)* enhances both sterility and RNAi defective phenotypes of the hypomorphic *drh-3* allele, suggesting that ELLI-1 functions with or in parallel to components of the CSR-1 complex.

CSR-1 pathway components share both Ego (Enhancer of Glp-One sterility) and Ekl (Enhancer of Ksr-1 Lethality) phenotypes [5,11,22,25,50]. *elli-1* RNAi failed to enhance *glp-1(bn18ts)* sterility at the permissive temperature of 20°, and there was no difference in rod-like larval lethality with *elli-1* RNAi in *ksr-1* mutants (S2 Table). Lethality in *ksr-1* mutants is attributed to a defect in excretory duct cell specification, a maternally contributed but somatic cell fate. While it is easy to over interpret negative results showing the lack of *glp-1* and *ksr-1* enhancement, these observations suggest that ELLI-1’s function with the CSR-1 complex is limited to a subset of CSR-1’s targets.

Another characteristic of *csr-1* is an RNA pooling defect in the germline of adults. This phenotype was proposed as a consequence of losing *csr-1* mRNA slicing activity, causing transcripts to accumulate that would normally be degraded [12]. Later, RNA expression in germlines dissected from *csr-1* RNAi-depleted animals failed to support this hypothesis; in contrast, mRNA targets of CSR-1-22G siRNAs in adult hermaphrodites were the least likely transcripts to change after a six-fold depletion of *csr-1* [13], correlating with previous findings from tiling array expression in *csr-1* mutants [4]. While some fine tuning of a fraction of CSR-1’s >4000 target mRNAs has now been reported in whole worms [14], the bulk of these targets show no change in dissected germlines when *csr-1* is compromised [13]. To see if *elli-1* mutants also have this same RNA pooling defect, ten dissected germlines from wild-type, *csr-1(fj54)*, and *elli-1(sam3)* animals were stained with the RNA dye SYTO14. While only 1/10 *wild-type* germlines showed RNA pooling, 10/10 *csr-1* (p = 0.0001) and 8/10 *elli-1* (p = 0.0054) germlines showed pooling in the central rachis and around the nuclear periphery of germ cells, although pooling in *csr-1* is more pronounced (Fig 6C). Since ELLI-1 does not contain mRNA slicing domains like CSR-1, the pooling defect in the germline of *elli-1* mutants, and potentially *csr-1* mutants, may instead reflect defects in mRNA shuttling and dispersal rather than changes in mRNA levels.

## Discussion

Based on EMS mutagenesis frequencies [51], we expected to get up to two loss-of-function mutations in each known component of the CSR-1 complex. This expectation came very close to our observation. A more saturated screen is likely to identify one or two more genes, but the ten alleles obtained in these five loci demonstrate both the specificity of the enlarged P-granule phenotype and the central role of the CSR-1 complex in the *C*. *elegans* germline.

We found that *elli-1* enhances RNAi efficacy and sterility in a hypomorphic *drh-3* allele, suggesting that ELLI-1 functions as part of or in parallel to the CSR-1 complex. The lack of overlap between *elli-1* and *csr-1* gene expression profiles better supports the latter possibility, and/or suggests that ELLI-1 and CSR-1 impinge on common targets post-transcriptionally to promote brood size, fertility, and RNAi efficacy. By residing on the nuclear periphery and extending the nuclear pore complex environment into the cytoplasm [52–55], P granules are well positioned to receive transcripts as they exit the nucleus, most of which then transit through P granules to reach the cytoplasm [56]. ELLI-1 is diffuse in the germ plasm (not enriched or excluded from P granules), but some ELLI-1 foci appear to dock next to P granules. One possibility is that CSR-1 shuttles its targets through P granules and into the cytoplasm to be translated (Fig 6D). ELLI-1 may be assisting in this process, although ELLI-1 does not contain known RNA-binding domains so a potential interaction with RNA may be indirect or through its predicted disordered region. The RNA pooling defects observed in *elli-1* and *csr-1* mutants could be a consequence of mRNA getting stuck in P granules and detached cytoplasmic RNPs. Cytoplasmic RNA distribution has recently been shown to drive phase separation of P-granule components [57–59], and it is worth further testing whether ELLI-1 and the CSR-1 complex function to disperse germline transcripts to antagonize phase separation, keeping P-granule size in check.

There are notable differences between *elli-1* and the four other genes obtained in this screen despite the indistinguishable P-granule phenotype that suggest ELLI-1 impinges on just a subset of CSR-1 targets. First, *elli-1* does not appear to enhance *glp-1* or *ksr-1* lethality. Second, *elli-1* mutants can be maintained as homozygotes with only minor impacts on brood size, while null alleles of *csr-1*, *drh-3*, *ego-1* and *ekl-1* must be maintained over a balancer. We expect that truncations in the two *elli-1* alleles obtained from the screen, as well as the two generated using CRISPR/Cas9, cause a complete loss-of-function as *elli-1* RNAi phenocopies the enlarged P-granule defect and low frequency of embryonic lethality. The hypomorphic *drh-3* allele obtained in this screen can also be maintained as a homozygote at permissive temperatures while exhibiting enlarged P granules. These results suggest that P-granule defects in CSR-1 pathway mutants can be disassociated from what is causing their embryonic lethality. Interestingly, the enzymatic slicing activity of CSR-1 was recently shown to fine tune expression in oocytes to support early embryogenesis; however, mutations introduced into CSR-1 to inhibit its slicing activity still cause embryonic lethality but were reported not to disrupt P granules [14]. Therefore, the CSR-1 pathway is likely to have both mRNA-slicing and non-slicing roles.

A final difference is that *elli-1* alone does not have RNAi defects like CSR-1 pathway mutants. This is important because while CSR-1 binds 22G RNAs, most evidence suggests that these siRNAs are not the product of exogenous RNAi; therefore, it has been proposed that defective transgene silencing and the Rde phenotypes of *csr-1* and *ego-1* are attributed to compromised P granules and the Rde phenotype of *pgl-1* [4]. Our results make this less likely as the P-granule phenotypes of *elli-1* and *csr-1* are indistinguishable in the adult germline, suggesting that components of the CSR-1 complex play a more direct role in exogenous RNAi. Because germline defects resulting from partial loss of the CSR-1 pathway are enhanced by *elli-1*, we conclude that ELLI-1 functions with the CSR-1 pathway to modulate RNAi activity, P-granule accumulation, and post-transcriptional expression in the germline.

## Materials and methods

### Strain maintenance

*C*. *elegans* strains were maintained as per standard protocols [60]. See S3 Table for list of strains used. N2, ZT3, TH206, MT8677 and the CB4856 Hawaiian isolate were obtained from the Caenorhabditis Genetics Center (CGC). EL44 was a gift from Eleanor Maine at Syracuse University. Remaining strains were generated in this study and are available upon request.

### Screen design

EMS mutagenesis was performed on TH206 worms using the standard protocol [61]. Two-thousand F1 progeny were cloned to individual plates, and F2 grandchildren were screened under a Leica M165FC fluorescence stereomicroscope for enlarged, bright PGL-1::GFP granules. This screen was done in parallel with a now published screen looking for somatic PGL-1::GFP expression [35]. To quantify P-granule size, cross-sections of the surface of ten germlines/strain were acquired with a 63X objective and fixed exposure conditions, and a fixed threshold was used to outline and measure the area of P granules using ImageJ.

### Mapping

CB4856 (Hawaiian) males were crossed into mutant strains. F1 cross progeny were picked to new plates, and on average 500 F2s worms with the enlarged P-granule phenotype were handpicked from each cross and pooled for whole genome sequencing [35,62]. Samples were sequenced on Illumina HiSeq2500 and NextSeq systems. The CloudMap pipeline was used to analyze mutant genome sequences, obtain map data, and find mutations as previously described [36].

### *csr-1* complementation

ZT3 males were crossed into DUP9, DUP12, DUP34, and DUP36 hermaphrodites. At least 15 male cross progeny (lacking the *nT1[qIs51] myo-2*::*GFP* balancer), were examined for the bright and enlarged P-granule phenotype. All male cross progeny without *myo-2*::*GFP* from DUP34 and DUP36 hermaphrodites had the phenotype, while male cross progeny without *myo-2*::*GFP* from DUP9 and DUP12 did not.

### CRISPR strain construction

A co-CRISPR technique was used to recreate mutation alleles as described [63]. A 19 base pair insert (TGAGACGTCAACAATATGG) was cloned into pJW1219 to direct Cas9 cleavage of *rol-6* (pDU58), and an 86 base pair Ultramer (IDT) was used as a homologous repair template to introduce the dominant *rol-6d* mutation (GTTAAACTTGGAGCAGGAACCGCTTCCAACCGTGTtcGgtGcCAgCAgTAcGGAGGATATGGAGCCACTGGTGTTCAGCCACCAGC). To create the *elli-1(sam21)* and *elli-1(sam22)* alleles, these 19 base pairs (ATATCCACATCTTACCGGG) were cloned into pJW1219 to direct cleavage (pDU59), and repaired with (ttatttcagATCTGCCAAACCAGGCCAAAAGTGCCGCCaGGcAAaATGTGaATATTCGAAAGTCGAACGATTTACGACGAGAATGG). To create the *drh-3(sam27)*, these 20 base pairs (GAATGATCCAGCTAATCGAG) were cloned into pJW1219 to direct cleavage (pDU57), and repaired with the 60 base pair oligo (AATGAAGAATGATCCAGCTAATCGAGC**a**GCT**t**TCTACTTTTTGGATAAGAACTGGCCAGA). 50ng/ul of pDU58 and 20ng/ul of the rol-6d repair oligo were co-injected with 50ng/ul of pDU59 or pDU57 and 20ng/ul of the corresponding repair oligo. Rolling F1 progeny were cloned, and their non-rolling progeny were genotyped for the corresponding mutations.

The GFP-SEC technique was used to attach C-terminal GFP::3xFLAG to endogenous *glh-1*, *elli-1*, and *pgl-1* as described [41]. To direct *glh-1* cleavage, (TCCCTCAAGATGAAGAAGGC) was inserted into pJW1219 (pDU69). To direct *elli-1* cleavage (TCATGCTCACGATGACGAT) was inserted into pJW1219 (pDU60). To direct *pgl-1* cleavage (GGTGGTTACGGGGGTCGTGG) was inserted into pJW1219 (pDU70). 500 base pairs of both 5’ and 3’ flanking sequences from *glh-1* (pDU67), *elli-1* (pDU68), and *pgl-1* (pDU73) were cloned into pDD282 to create homologous repair plasmids.

### RNAi feeding

RNAi feeding constructs were obtained from the Ahringer library. The L4440 plasmid in HT115 bacteria was used as the RNAi control. *elli-1* RNAi was performed on L4 worms in three biological replicates, and the PGL-1::GFP phenotype was observed in F1 progeny.

To look for enhancement of *glp-1(bn18ts)*, approximately 60 L1 stage EL44 worms were placed on each of five control (empty L4440 vector) or five *elli-1* RNAi plates at 20°C and scored for a clear sterile phenotype three days later. To compliment these findings, approximately 60 L1 stage worms of each EL44 *glp-1(bn18ts)*, DUP67 *elli-1(sam3)*, and DUP119 *glp-1(bn18ts); elli-1(sam3)* double mutants grown at 20°C were examined for *glp-1* like sterility. To look for enhancement of *ksr-1(n2526)*, control and *elli-1* RNAi was performed as previously described [50].

To assay RNAi enhancement, approximately 60 L1s were placed on each of five *his-44* RNAi feeding plates for each strain, and animals arrested during larval development were scored 3 days later [35,64].

### Fosmid rescue

DUP9 was injected with the fosmid WRM0610dF01 (1.5ng/ul) to create DUP61. All injections used the *myo-3p*::mCherry coinjection marker pCFJ104 (10ng/ul) [65].

### Fluorescent in situ hybridization (FISH)

A Stellaris FISH probe (Biosearch Technologies) was designed for *elli-1* with CAL Fluor Red 610. Whole-worm FISH was performed as previously described [13,66]. Images were acquired and deconvolved using Leica AF6000 acquisition software on an inverted microscope (Leica DMI6000B) with a cooled CCD camera (Leica DFC365FX) and a Leica 40x air objection with a 0.5 um z-stack. All white scale bars throughout the paper are 20 microns in length.

### SYTO14 RNA stain

Wild-type, *csr-1(fj54)*, and *elli-1(sam3)* germlines were dissected and stained with SYTO14 to visualize RNA as described [12].

### Immunocytochemistry

Dissected germlines were fixed using methanol/acetone [67], and stained with rabbit anti-PGL-1 or rabbit anti-GLH-1 polyclonal antibodies, and co-stained with monoclonal mAb414 as previously described [68]. To examine *elli-1*::*gfp*::*3xFLAG* expression and colocalization with PGL-1, dissected germlines and embryos were fixed with methanol acetone and co-stained with rabbit anti-PGL-1 [68] or rabbit anti-CGH-1 [69] and a 1:1000 dilution of M2 anti-FLAG monoclonal antibody (Sigma-Aldrich-F1804), followed by staining with anti-rabbit Alexa 594 and goat anti-mouse Alexa 488 conjugated secondaries and DAPI. To quantify PGL-1 expression in Fig 1C, dissected germlines were fixed and stained with rabbit anti-PGL-1, and a pixel intensity was measured in a fixed region on interest in pachytene germ cells; then normalized to the parental control (n>18 germline images per strain).

### Brood size assay

For each strain, brood sizes were counted from each of ten L4 animals after placing them at 20°C and 25°C.

### Embryonic lethality

For each strain, six adult worms were placed on a plate and allowed to lay for 5 hours. The number of embryos on each plate was counted, and unhatched embryos were counted the following day.

### Microarray

For each of the four biological replicates of TH206 and DUP56 strains, synchronized L1 larvae were seeded on 10 plates, grown at 20°C, and young adults were collected (corresponding to 90% with a vulva and no or few eggs). Young adults were washed three times, pelleted, and frozen. Total RNA was extracted with Trizol Reagent and ran through an RNA Stable kit (Biomatrica), which was submitted for expression analysis. OakLabs (www.oak-labs.com) prepared samples and hybridized them on the 8x60K Agilent ArrayXS *C*. *elegans* tiling array, and performed gene expression analysis (GEO accession number GSE82322). These microarrays also contain several thousands of piRNA probes, but as there was no significant change in piRNA levels, these probes were excluded from S1 Table. qRT-PCR validation of *pgl-1*, *pgl-3*, and *glh-1* expression in N2 and DUP67 worms as described [12].

## Supporting information

S1 Table *elli-1* microarray expression. (XLSX)

S2 Table Analysis of Ego and Ekl phenotypes. (DOCX)

S3 Table List of strains used in this study. (DOCX)
