# Supplementary material for: ELLI-1, a novel germline protein, modulates RNAi activity and P-granule accumulation in Caenorhabditis elegans
Source: PLoS Genet. 2017 Feb 9;13(2):e1006611. doi: 10.1371/journal.pgen.1006611 (PMC5325599; doi:10.1371/journal.pgen.1006611)
Supplement: S2 Table — (DOCX) [file pgen.1006611.s002.docx]

**Table S2**

**Analysis of Ego and Ekl Phenotypes**

| **Strain Name** | **Genotype** | **RNAi** | **replicates** | **Sterile** | **SD** | **Rod Lethal** | **SD** |
| --- | --- | --- | --- | --- | --- | --- | --- |
| **N2** | *wild type* | Empty Vector | 5 | 0.00% | 0.00 |  |  |
|  | “ | *elli-1* | 5 | 0.00% | 0.00 |  |  |
| **EL44** | *glp-1(bn18ts)* | Empty Vector | 5 | 0.68% | 0.83 |  |  |
|  | “ | *elli-1* | 5 | 1.11% | 0.99 |  |  |
| **EL44** | *glp-1(bn18ts)* | none/OP50 | 1 | 1/64 | - |  |  |
| **DUP67** | *elli-1(sam3)* | none/OP50 | 1 | 0/75 | - |  |  |
| **DUP119** | *glp-1(bn18ts); elli-1(sam3)* | none/OP50 | 1 | 1/82 | - |  |  |
| **N2** | *wild type* | Empty Vector | 10 |  |  | 0.00% | 0.00 |
|  | “ | *elli-1* | 10 |  |  | 0.00% | 0.00 |
| **MT8677** | *ksr-1(n2526)* | Empty Vector | 10 |  |  | 1.70% | 1.64 |
|  | “ | *elli-1* | 10 |  |  | 1.57% | 1.66 |
