# Supplementary material for: ELLI-1, a novel germline protein, modulates RNAi activity and P-granule accumulation in Caenorhabditis elegans
Source: PLoS Genet. 2017 Feb 9;13(2):e1006611. doi: 10.1371/journal.pgen.1006611 (PMC5325599; doi:10.1371/journal.pgen.1006611)
Supplement: S3 Table — (DOCX) [file pgen.1006611.s003.docx]

**Table S2**

**Worm strains used in this study**

| **Strain Name** | **Genotype** | **Description** |
| --- | --- | --- |
| N2 | Wild Type |  |
| CB4856 | Hawaiian Isolate | Used for SNP mapping |
| TH206 | *ddEx16[pgl-1::PGL-1::TY1::EGFP::3Xflag + Cbr-unc-119(+)] I* | *** *ddEx16* is actually integrated on chromosome I [35] |
| DUP8 | *ddEx16 drh-3(sam2) I* | 1X outcrossed |
| DUP9 | *ddEx16 I; elli-1(sam3) IV* |  |
| DUP11 | *[ddEx16 (sam5)]/[ddEx16 +] I* |  |
| DUP12 | *ddEx16 I; [elli-1(sam6)/+] IV* | Homozygous lethal due to linked mutation |
| DUP13 | *[ddEx16 (sam7)]/[ddEx16 +] I* |  |
| DUP17 | *ddEx16*[*pgl-1::PGL-1::TY1::EGFP::3Xflag + Cbr-unc-119(+)*] I | TH206 outcrossed 2X to N2 |
| DUP22 | *[ddEx16 ego-1(sam14)]/[ddEx16 +] I* |  |
| DUP23 | *ddEx16 I; [csr-1(sam15)/+] IV* |  |
| DUP24 | *[ddEx16 ego-1(sam16)]/[ddEx16 +] I* |  |
| DUP26 | *ddEx16 I; [csr-1(sam18)/+] IV* |  |
| DUP27 | *[ddEx16 ekl-1(sam19)]/[ddEx16 +] I* |  |
| DUP34 | *ddEx16 I; [csr-1(sam15)(IV)/nT1[qIs51](IV;V)]* | Balanced *csr-1(sam15)* |
| DUP36 | *ddEx16 I; [csr-1(sam18)(IV)/nT1[qIs51](IV;V)]* | Balanced *csr-1(sam18)* |
| DUP46 | *[ddEx16 ego-1(sam16)]/[ddEx16 dpy-5(e61)] I* | Balanced *ego-1(sam16)* |
| DUP56 | *ddEx16 I; elli-1(sam21) IV* | CRISPR to create same *sam3* + silent mutations |
| DUP57 | *ddEx16 I; elli-1(sam22) IV* | CRISPR deletion and frame shift in *elli-1* |
| DUP60 | *[ddEx16 ego-1(sam14)]/[ddEx16 dpy-5(e61)] I* | Balanced *ego-1(sam14)* |
| DUP61 | *ddEx16 I; elli-1(sam3) IV; samEx6(F20C5.3 fosmid + myo-3::mCh)* | *F20C5.3* fosmid injected into DUP9 to test rescue |
| DUP64 | *glh-1(sam24[glh-1::gfp::3Xflag]) I* | CRISPR GFP-SEC method used to tag *glh-1* with GFP |
| DUP67 | *elli-1(sam3) IV* | 4X outcrossed to N2, no GFP transgene |
| DUP68 | *drh-3(sam27) I* | CRISPR to create same *sam2* + silent mutations |
| DUP69 | *glh-1(sam24[glh-1::gfp::3Xflag]) I; elli-1(sam3) IV* | Created from DUP67 & DUP64 |
| DUP71 | *elli-1(sam28[elli-1::gfp::3Xflag]) IV* | CRISPR GFP-SEC method used to tag *elli-1* with GFP |
| DUP75 | *pgl-1(sam33[pgl-1::gfp::3Xflag]) IV* | CRISPR GFP-SEC method used to tag *pgl-1* with GFP |
| DUP76 | *drh-3(sam27) I; pgl-1(sam33[pgl-1::gfp::3Xflag]) IV* | Created from DUP68 & DUP75 |
| DUP79 | *drh-3(sam27) I; elli-1(sam3) IV* | Created from DUP67 & DUP68 |
| DUP98 | *patr-1(sam50[patr-1::gfp::3xflag]) II* | CRISPR GFP-SEC method used to tag *patr-1* with GFP |
| DUP119 | *unc-32(e189) glp-1(bn18ts) III; elli-1(sam3) IV* | Created from DUP67 & EL44 |
| EL44 | *unc-32(e189) glp-1(bn18ts) III* | From [20] |
| MT8677 | *ksr-1(n2526) X* | From [50] |
| ZT3 | *csr-1(fj54)(IV)/nt1[qIs51](IV;V)* | From [4] |
